# Learnings From the Implementation of an Electronic Human Resource Management System for the Health Workforce in Uttar Pradesh, India

**DOI:** 10.9745/GHSP-D-23-00312

**Published:** 2024-06-27

**Authors:** Sushant Jain, Vasanthakumar Namasivayam, Shivalingappa Halli, Shajy Isac, Marissa Becker, Mushahid Ali Khan, Vikas Gothalwal, James Blanchard, Pooja Pandey, Awadhesh Kumar Rawat, Ravi Prakash

**Affiliations:** a Institute for Global Public Health, Department of Community Health Sciences, University of Manitoba, Winnipeg, Canada.; b India Health Action Trust, Lucknow, Uttar Pradesh, India.; c Department of Medical Health & Family Welfare, Government of Uttar Pradesh, India.; d National Informatics Centre, Lucknow, Uttar Pradesh, India.

## Abstract

The state of Uttar Pradesh (UP), India, has one of the largest single public health systems globally, serving about 235 million people through more than 30,000 public health facilities with approximately 160,000 health personnel. Yet, the UP health system has a shortfall of public health facilities to meet the population’s needs, a shortage of clinical and nonclinical health personnel, inequitable distribution of existing health personnel, and low utilization of public health facilities. A robust and effective electronic human resource management system (eHRMS) that provides real-time information about the lifecycle of all health professionals in UP may aid in improving the health workforce, resulting in better health services and improved health outcomes. The Government of UP rolled out Manav Sampada, a comprehensive eHRMS that complied with global norms and requirements. We describe the implementation of Manav Sampada at scale and elaborate on key learnings and adoption strategies. Manav Sampada was based on key principles of integration and data-sharing with other digital systems, included functional components, a minimum dataset, used a lifecycle-based approach, and a workflow-based system, all of which acted to improve human resource data quality. The eHRMS emerged as a valuable tool for key stakeholders in reviewing worker performance, identifying skill-building needs, and allocating resources for training, leading to improved availability and equity in the distribution of a few critical cadres. The eHRMS in UP is well positioned to become an integral part of the Ayushman Bharat Digital Mission, the backbone of India’s integrated digital health infrastructure. Linking eHRMS to a planned beneficiary-centric unitized health service delivery system (capturing information at the individual level rather than the aggregate level) will enable the measurement of service delivery and quality, leading to improved workforce management.

## Abstract (precis)

Electronic human resource management systems can be used to improve the equitable distribution of the health workforce, contributing to increased availability of health services and improved health outcomes.

## Teaser

**Key Findings:**

- A personnel information system that was being used for human resource management in Uttar Pradesh was missing about 40% of the total health workforce in the state’s public health system and lacked critical components compared to global standards.
- The customization of an electronic human resource management system (eHRMS) aimed to ensure coverage of the health workforce, improve data quality, and increase the use of data for planning and decision-making.
- After the eHRMS roll-out, registration of the entire health workforce increased, vacancies of some critical cadres in community health centers and primary health centers decreased, interoperability with other health information systems increased efficiency and reduced duplication of effort, and data quality improved.

**Key Implication:**

- Policymakers should consider having an eHRMS for health personnel to improve the availability and equity in the distribution of critical cadres.

## INTRODUCTION

With an estimated population of 235 million, Uttar Pradesh (UP) is India’s most populous state, accounting for approximately one-sixth of the country’s population.^1^ As of May 2024, UP has approximately 160,000 health personnel (including contractual/temporary workers) belonging to both clinical and managerial/administrative cadres in the public health system distributed across 75 districts and 820 subdistrict administrative units. These health personnel are employed by the Government of UP (GUP) Department of Medical, Health and Family Welfare (DMHFW) in more than 30,000 public health facilities, including 25,728 subcenters, 3,645 primary health centers (PHCs), 964 community health centers (CHCs), and 107 district hospitals providing primary and secondary health care services to the population of UP, including 170 million people in rural areas.^2^

Despite the large number of public health facilities, they are inadequate to meet the population’s health care needs compared to the prescribed national norms defined by the Indian Public Health Standards based on the rural population. The shortfall of different types of public health facilities ranges between 42% and 51%.^3^ This shortfall is further compounded by the shortage of critical clinical and nonclinical human resources (HR). For example, about 25% of the sanctioned female health worker positions at subcenters and PHCs in rural areas are vacant.^3^ The most recent National Sample Survey data on the social consumption of health show that only 14% and 28% of the rural population in UP access public sector facilities for outpatient and inpatient (hospitalization cases excluding childbirth) services, respectively.^4^ This low utilization of services may be due to the HR shortage, inequitable distribution of existing human resources for health (HRH),^5^ and inadequate quality of services in public health facilities, in addition to other factors that affect the utilization of services. To help mitigate this situation, better workforce planning and management are of the utmost importance.

HR management, defined as the “integrated use of systems, policies, and practices that will provide the range of functions needed to plan, produce, deploy, manage, train, support, and sustain the workforce,”^6^ is critical to achieving improved health outcomes. The Human Resources Management Assessment Approach states “without a relatively solid foundation of up-to-date workforce information and baseline data, critical workforce planning decisions and targeted scale-up interventions become difficult if not impossible to project, implement, and measure with any great degree of accuracy.”^6^ The World Health Organization (WHO) also emphasizes the importance of having an electronic HR management system (eHRMS) that enables rapid aggregation and display of health workforce data for decision-making.^7^ Moreover, the HRH Action Framework places HR management systems at the center of achieving an improved health workforce, better health services, and better health outcomes.^8^ Considering UP’s large health workforce, vast geography with a rural context, and poor health outcomes, having an eHRMS becomes crucial to achieving improved health outcomes in UP.

An eHRMS enables rapid aggregation and display of health workforce data for decision-making.

### Readiness Assessment

In 2014, the Government of India provided additional funding to states to roll out an eHRMS.^9^ In 2016, the DMHFW, in collaboration with the Uttar Pradesh Technical Support Unit (UPTSU), conducted a readiness assessment for designing and implementing an eHRMS that would meet the global norms. UPTSU was established in 2013 by the University of Manitoba and India Health Action Trust based on a memorandum of understanding between the GUP and the Bill & Melinda Gates Foundation and provides technical support to the GUP to improve the population’s health and nutrition outcomes.

As part of this assessment, UPTSU conducted a literature review to guide the design, development, and implementation of the eHRMS. The WHO document, *Human Resources for Health Information System: Minimum Data Set for Health Workforce Registry,* which describes the concepts and functions of an eHRMS, served as a standard tool for the assessment.^7^ An eHRMS had to: (1) enable health workforce data interoperability with other health information systems (e.g., disease surveillance, patient management, supply chain and commodity); (2) include functional components such as migration, payroll, benefits, vacancy and recruitment, retirement, performance management, and training; (3) include a minimum dataset for the health workforce registry (e.g., identification number, name, contact information); and (4) be lifecycle based and person-centric.

**FIGURE 1:**
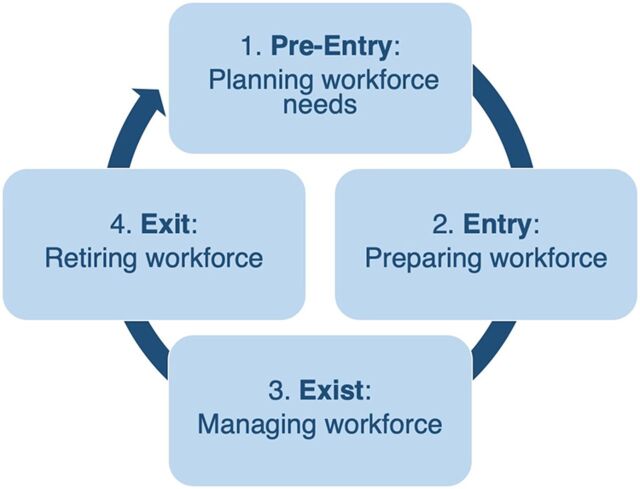
Four Major Workforce Functional Domains of the Health Workforce Lifecycle Adapted from World Health Organization 2015.^7^

During the readiness assessment, we found that the DMHFW had been using an eHRMS called the Personnel Information System (PIS) for more than a decade. After comparing this system to the WHO tool, the UPTSU identified the following gaps in PIS: (1) lacked unique identifiers for data elements (e.g., health facility) that would enable data interoperability with other systems within the broader health information system; (2) lacked critical modules (e.g., migration, training, and performance management; (3) did not include a few critical elements of the minimum dataset (e.g., birth history, citizenship, contact information, occupational category [a subset of employment status]), and data submission institution; and (4) was not lifecycle based (Figure 1).

Additionally, the PIS had only about 80,000 regular/permanent employees registered on the system compared to more than 140,000 employees (as of 2016) and did not include contractual/temporary workforce registration, missing about 40% of the total health workforce in the UP public health system available as of 2016. Per WHO norms, the PIS did not account for all health personnel in UP to qualify as a workforce registry due to inadequate coverage. An independent assessment of eHRMS of different states of India by the U.S. Agency for International Development stated that although the PIS had been in place for more than 8 years, it was considered at “an early stage of implementation” not warranting further detailed analysis, unlike 14 other states that had eHRMS considered to have been “established” and taken up for detailed study.^10^ Hence, considering the digital architecture and implementation status of PIS, GUP decided that it could not be upgraded to meet the global norms for eHRMS and warranted a new system.

To use a robust system that met global norms, the UPTSU recommended Manav Sampada (meaning human wealth or human assets), an eHRMS developed by the National Informatics Centre (NIC) of the Government of India (https://ehrms.nic.in/). Manav Sampada had the following features: (1) allowed interoperability with other health information systems; (2) included most of the functional components of HR-related processes and opportunities for expansion; (3) included a minimum dataset for the database of all health personnel; and (4) allowed tracking of any personnel in the system until the end of life or departure from the department due to retirement, resignation, or termination.

**FIGURE 2:**
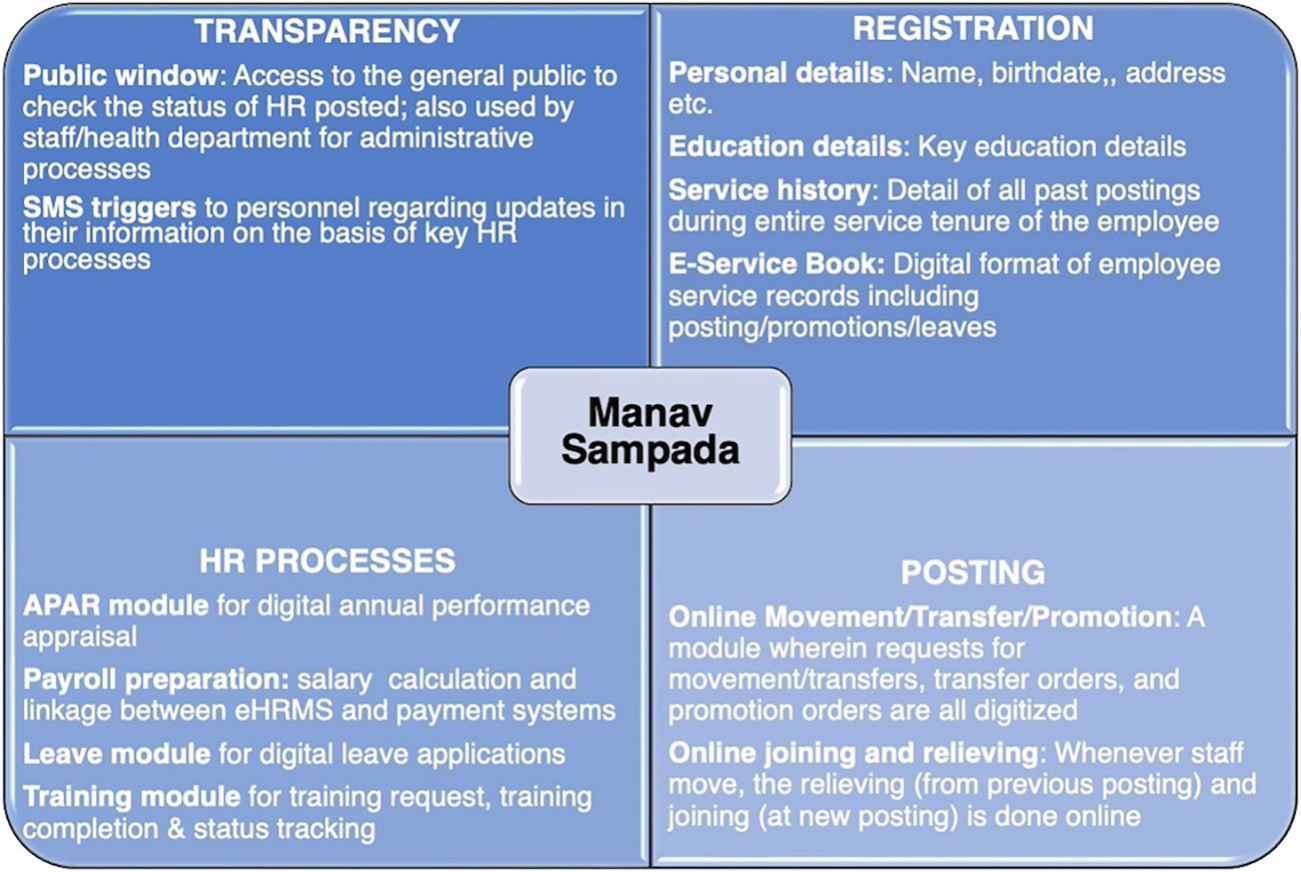
Key Features/Modules of the Manav Sampada eHRMS Abbreviations: APAR, annual performance appraisal report; eHRMS, electronic human resource management system; HR, human resource.

The DMFHW accepted the UPTSU’s recommendation and requested support in the system’s roll-out. UPTSU played a critical role in designing/customizing and implementing Manav Sampada to meet the DMHFW’s requirements (Figure 2).

## IMPLEMENTATION AND ADOPTION STRATEGIES OF MANAV SAMPADA

Literature on key design principles and implementation strategies of an eHRMS at scale is limited. Most studies on eHRMS have focused on the availability, quality, and use of eHRMS data.^11^^–^^13^ We describe the approaches undertaken to implement a robust eHRMS at scale and its adoption by GUP stakeholders. We also summarize the key strengths, limitations, and lessons learned during the implementation and adoption process. The lessons can be applicable for adoption in other states in India and in other low- and middle-income countries.

The implementation and adoption strategies aimed to ensure coverage, quality, and utilization of eHRMS data. The eHRMS was designed to be interoperable, already had key functional components, included a minimum dataset, used a lifecycle-based approach, bound eHRMS registration to salary release, used a workflow-based design, included measures to improve data quality, provided value for key stakeholders, and used practical approaches regarding data entry.

The implementation and adoption strategies aimed to ensure coverage, quality, and utilization of eHRMS data.

### Interoperability

The system was designed and customized to follow meta-data standards, enabling integration and data-sharing with the following health information systems.

- The HMIS aims to capture the aggregated monthly service delivery outputs from all public health facilities, requiring up-to-date data on the availability of HR.^14^ The HR data required in HMIS was directly linked to eHRMS, preventing duplication of effort and reflecting updated HR availability status.
- UP Ke Swathya Kendra^2^ is a geographic information system-based application that serves as the public health facility registry and integrates facility-based information regarding HR, logistics, and service outputs. Due to the interoperability of eHRMS, this integration was easily achieved by fetching up-to-date personnel information from the eHRMS.
- Financial management system: The eHRMS was designed and customized to enable the preparation of payroll and seamless data exchange with the payment gateway.

### Key Functional Components

Manav Sampada had all the key functional core components as defined by WHO except for the workforce production module (discussed further in the challenges section) and satisfied the key requirements of DMHFW.

### Minimum Dataset

Basic registration of personnel should ideally contain at least the minimum data for the workforce registry as outlined by WHO^7^ (Box). Although Manav Sampada had the option of more than 100 data points, it was customized to make only those 10 data elements mandatory, simplifying the registration process. Because DMHFW was sure about the licensing and certification of its employees, due to robust verification at the time of entry into the public health system, it was decided to make the subelement of professional license/certification non-mandatory.

BOX Minimum Dataset Required for Registration of Health Workforce in Manav Sampada

1. Identification number
2. Full name
3. Birth history
4. Citizenship, country of residence, and language
5. Address
6. Contact information
7. Education, professional license,^a^ and certification^a^
8. Employment status
9. Employment address
10. Data submission institute

^a^Not mandatory data.

### Lifecycle-Based Approach

The “lifecycle” of health personnel includes their registration and licensure, recruitment, deployment, and actions taken to promote them and improve their productivity, their transition within the system or out of it, and their eventual exit from the workforce (through retirement, migration, or death).^15^ eHRMS in UP was developed following this lifecycle-based approach.

- **Deployment/distribution to a specific posting:** All new recruits must first be registered on Manav Sampada with their employment order mandatorily uploaded to the system.
- **Transition to a complete digital system to capture movements:** To track internal movements due to transfers/relocation, Manav Sampada captures “online relieving” from the current posting and “online joining” at the new posting. This ensures that the database and current posting office of an employee are updated systematically, and no manual data entry is required to keep the system up to date. To ensure compliance, DMHFW released orders that any transfers done outside Manav Sampada would be considered “null and void.”
- **Promotion:** Manav Sampada is used to manage employees’ promotion process.
- **Exit:** The system manages the exit of an employee due to retirement, resignation, and study leave and has provisions to facilitate exit and retirement benefits management, as well as management in the case of the death of personnel.

### Binding Registration to Salary Release

One of the DMHFW’s first targets for the eHRMS was 100% enumeration of all health personnel. Although registering new recruits was mandated, ensuring registration of all existing in-service personnel required a mechanism to ensure compliance. DMHFW linked Manav Sampada with the state-run digital treasury system to check registration status in eHRMS before payment of salaries. This ensured that only those employees who were registered on Manav Sampada (and also registered in the state-run digital treasury system under the same office/district) were eligible for salary payment at the end of each month. By implementing this strategy, DMHFW was able to achieve 100% enumeration of more than 140,000 health personnel within 6 months of the eHRMS roll-out. This also helped the DMHFW identify and take action against employees who absconded or were absent long term.

### Workflow-Based Design

In its simplest form, a workflow may be described as a set of tasks following a predefined sequence of rules and processes. In the world of digital systems, it can be defined as “the computer-assisted implementation of a business process,” which includes the graphical definition or description of how to perform the process and the engine to execute it.^16^ In Manav Sampada, the workflow was customized and designed to replicate real-world workflow-based data generation and verification for the following processes.

- eHRMS enables the application, approval, and acceptance of the transfer process involving multiple stakeholders to only enter data related to their work, as data flows in workflow-based sequence to the concerned stakeholders, eliminating redundancies in duplicating data entry efforts.
- The workflow-based system of the performance appraisal process from self-appraisal to appraisal by a reporting officer to acceptance by a reviewer enables the timely completion of the annual appraisal process.
- The workflow-based system of the training module enables training calendar preparation, application for training, nomination, tracking of completion status, and generation of an e-certificate.

### Measures to Improve Data Quality

The lack of routine data use at all levels leads to the poor availability and poor quality of data in low- and middle-income countries.^17^ Although the DMHFW achieved 100% enumeration of the workforce, there was a lack of confidence in the data quality. Several initiatives were done with the UPTSU support to improve data quality through “data cleaning exercises.” Throughout 2017 and 2018, massive camps and elaborate processes were set up for these exercises. However, these efforts yielded minimal success, leading to the improvement in the quality of only existing data. However, the HR data are dynamic, requiring continual updating of events.

The UPTSU realized that stakeholders’ use of data for decision-making improves the data quality over time and leads to more sustainable and tenable results than sporadic efforts by external data operators. In 2019, the DMHFW started using eHRMS data for deployment-related decisions based on HR gaps identified for doctors’ and nurses’ cadres, leading to the strengthening of data quality to the extent that the accuracy of data points for doctors, nurses, and midwives is currently more than 90%. As part of data-based HRH system strengthening, the quality of the data points was occasionally assessed internally by the Program Management Unit (PMU), a government-level institutional mechanism that regularly monitors the eHRMS implementation. For example, in 2021, to ensure that a medical officer (MBBS doctor) was posted in each of the state’s more than 3000 PHCs, the PMU established a call center that called the mobile numbers of all doctors posted at PHCs and listed in the eHRMS to confirm/update their place of posting. Through this effort, it was found that more than 90% of the phone numbers were correct. This method improved the accuracy of the current place of postings of PHC doctors. In 2022–2023, a QR code-based “experience certificate” was required to be generated to provide additional marks (as per state policy) to contractual auxiliary nurse-midwives who wanted to apply for regular/permanent vacant posts. The eHRMS could generate the experience certificates for more than 95% of the contractual auxiliary nurse-midwives, along with the correct duration of their experience.

The DMHFW started using eHRMS data for deployment-related decisions based on HR gaps identified for doctors’ and nurses’ cadres, leading to the strengthening of data quality.

### Value for Key Stakeholders

Faster adoption of eHRMS was aided by designing modules that are useful for different stakeholders, such as employees, personnel division, and health administrators at various levels.

- Performance appraisal module: Before the eHRMS, doctors’ performance appraisal reports used a paper-based system that lacked transparency and made it difficult to compile the reports, often leading to delayed promotions that affected employees’ morale. To overcome this challenge, a customized module for the online annual performance appraisal report (APAR) was developed and successfully implemented for doctors, nurses, and dental cadres. This enabled the timely completion of the appraisal cycle and aided a faster promotion process, benefitting both employees and health administrators.
- Leave module: The process for employees applying for different types of leave that required approvals at various levels was digitized on Manav Sampada along with a mobile application. This module enables employees to apply for leave and track the approval status and leave balance in real time, notifies the approval authority of the pending applications and leave balance of individual employees, and provides the administrators with the status of facility-wide employee availability for the leave application period, aiding appropriate decisions to ensure the continuity of health services.

These modules were tactically introduced as initial modules expecting faster adoption by the employees. This helped us in implementing other modules, such as the training module, which may not have had the same initial buy-in from employees.

### Practical Approaches Regarding Data Capture

While filling in information regarding their in-service trainings, employees often had difficulty recalling the details of the past trainings that they may have undergone since joining. Considering this, we prioritized capturing in-service training data only prospectively by creating a workflow-based training module so that all future trainings would be successfully captured. The training module in Manav Sampada ensures that 100% of future staff trainings is systematically captured to improve service availability and quality.

## REFLECTIONS ON ACHIEVEMENTS, CHALLENGES, AND THE WAY FORWARD

Health systems can only function with health workers; improving health service coverage and health outcomes is dependent on their availability, accessibility, acceptability, and quality.^18^ The roll-out of an eHRMS in UP has led to an improvement in HR planning and thus may (directly or indirectly) have contributed to an improvement in the effective coverage of HR in multiple ways. For example, Manav Sampada’s roll-out made the critical HR gaps noticeable at the facility level across the state on a continuous basis, prompting action by the health administrators to close the availability gaps.

### Improved Access to Services and Need-Based Assignment of Critical Human Resources

According to the Rural Health Statistics data, from 2015 to 2021, vacancies of specialists at CHCs in UP decreased from 77% to 69% and vacancies for doctors at PHCs in rural areas decreased from 51% to 35%.^19^ Accessibility of lifesaving comprehensive emergency obstetric and newborn care closer to the community was enhanced using eHRMS by pairing and rational distribution of complementary skills of surgeons and anesthetists at the CHCs.

Accessibility of lifesaving comprehensive emergency obstetric and newborn care closer to the community was enhanced using eHRMS.

With the use of eHRMS, access to critical HR has improved. Because detailed facility-level data were now available, in 2020, GUP was able to assign the posting facility directly compared to the earlier practice of assigning only a posting district and having the district authorities decide the posting facility. This is believed to have led to an increase in access to doctors at the primary health care level. In 2018, only 2,947 doctors were posted at PHCs, and in 2023, the number increased to 4,743, a 61% increase in the last 5 years.

### User Satisfaction

The eHRMS has gradually evolved as a more effective digital platform, proving to be increasingly valuable to employees in meeting their requirements. In 2019, the online APAR module was implemented for all doctors, and 8,667 of these doctors submitted their appraisals online and in a timely manner. Based on this experience, the online APAR module was later expanded to dental, clerical, and nursing cadres. Based on the successful adoption and increased usage, in 2023, the number of staff who submitted their online APAR has increased to 11,256, a 30% increase in 4 years. Additionally, the leave module, which was implemented at the beginning of 2021, saw a 7-fold increase (from 33,000 to 241,000) in the number of “total leaves applied” from 2021 to 2023, signaling a significant increase in the use of the eHRMS modules by end users.

### Implementation Challenges

The implementation of eHRMS faced numerous challenges. The DMHFW witnessed multiple changes in leadership, necessitating constant advocacy for roll-out. Because the original software belonged to another state, NIC-UP faced technical challenges in adoption and customization. Although the PMU was set up to ensure government involvement, building the same level of ownership at the substate level was difficult during the initial period. The workforce production module was not included in the roll-out because the Department of Medical Education, which is responsible for the production of the medical workforce, is separate from the DMHFW.

### Way Forward

Going forward, the Government of India’s requirement to create national health professional identification numbers under the Ayushman Bharat Digital Mission, a national initiative that aims to create the backbone necessary to support India’s integrated digital health infrastructure, will be facilitated through application programming interface linkages of eHRMS with Ayushman Bharat Digital Mission.^20^ This enables GUP to measure progress in creating national identification numbers against clearly identified denominators.

UP is rolling out a digital system that can capture services delivered to each citizen. Linking eHRMS to this beneficiary-centric unitized health service delivery system will enable the measurement of service delivery by each HR. The learnings from the roll-out of eHRMS in UP can serve as guidance for policymakers and program managers in designing, planning, and implementing other eHRMSs at large scale.

### Transition and Sustainability of Manav Sampada

In the field of public health, sustainability has been defined as the capacity to maintain program services at a level that will provide ongoing prevention and treatment for a health problem after the termination of major financial, managerial, and technical assistance from an external donor.^21^ There are many examples of nongovernmental organization-driven programs/interventions not being sustained after the termination of external funding. To avoid this situation, the UPTSU and DMHFW set up the PMU at the beginning of the eHRMS roll-out to make critical decisions with an assured budget and human resources.

The NIC-UP ensured technology sustainability and local customization requirements for DMHFW by creating a separate version of the Manav Sampada database for DMHFW, as multiple GUP departments use Manav Sampada, and setting up its own data/application hosting systems.

### Limitations

This article does not cover the information technology-related requirements of an eHRMS, including the personnel requirements for developing and maintaining the application or the infrastructure/network requirement for hosting and maintaining the system. The budgetary requirement for implementing eHRMS has not been dealt with in this article as the Manav Sampada software was provided free of cost by NIC-Government of India to UP. Additionally, in the absence of a control group, it is difficult to attribute the improvements in HR management to the implementation of the eHRMS in UP. However, the absence of parallel paper-based systems, universal coverage of HRH, increasing use of key modules by employees, timeliness of key HRH processes, such as promotions and APAR, and use of its data by multiple stakeholders serve as a good proxy of the need and usefulness of the system. The impact assessment of the eHRMS in improving health outcomes needs to be studied further. However, existing frameworks^6^^–^^8^ underline the critical role of eHRMS in achieving health outcomes.

## CONCLUSION

We believe that implementation and adoption strategies based on the principles highlighted can help in effectively rolling out an eHRMS for health personnel in various contexts. Hence, states in India and other low- and middle-income countries (and their states/provinces) that are planning to roll out eHRMS may want to adopt these strategies and principles.

## Notes

**Peer Reviewed**

**First published online:** June 3, 2024
